# Impact of renal insufficiency on mortality in patients with ST-segment elevation myocardial infarction treated with primary percutaneous coronary intervention

**DOI:** 10.1186/1471-2261-14-15

**Published:** 2014-02-07

**Authors:** Jonas Emil Sabroe, Per Thayssen, Lisbeth Antonsen, Mikkel Hougaard, Knud Nørregaard Hansen, Lisette Okkels Jensen

**Affiliations:** 1 Department of Cardiology, Odense University Hospital, Sdr. Boulevard 29, 5000 Odense, Denmark

**Keywords:** ST-segment elevation myocardial infarction, Renal insufficiency, Mortality

## Abstract

**Background:**

Chronic kidney disease is associated with increased risk of mortality. We examined the impact of moderate and severe renal insufficiency (RI) on short- and long-term mortality among unselected patients with ST-segment elevation myocardial infarction (STEMI) treated with primary percutaneous coronary intervention (PCI).

**Methods:**

From January 1, 2002 to December 31, 2010 all patients with STEMI treated with primary PCI were identified. The hazard ratio (HR) for death was estimated using a Cox regression model, controlling for potential confounders. RI was defined as creatinine clearance (CrCl) < 60 mL/min (moderate RI: CrCl ≤30 < 60 mL/min and severe RI: CrCl < 30 mL/min).

**Results:**

The study cohort consisted of 4,116 patients of whom 898 (21.8%) had RI and 3,218 (78.2%) had a CrCl ≥ 60 mL/min. Compared to patients without RI, patients with RI were older, more often female and more likely to have diabetes mellitus, hypertension and to present with a higher Killip class.

Among patients with a preserved kidney function and patients with RI, 30-day all-cause mortality was 3.5% vs. 20.9% (log-rank p < 0.001); 1-year all-cause mortality was 5.7% vs. 29.4% (log-rank p < 0.001); 5-year all-cause mortality was 13.4% vs. 47.4% (log-rank p < 0.001). Moderate and severe RI were associated with higher 1-year mortality compared to patients with a preserved renal function (CrCl ≤30 < 60 mL/min: adjusted HR 2.71 [95% CI 2.09-3.51], p < 0.001), and (CrCl < 30 mL/min: adjusted HR 7.09 [4.82-10.44], p < 0.001).

**Conclusion:**

In unselected STEMI patients treated with primary PCI, moderate and severe RI were associated with increased risk of mortality.

## Background

Cardiovascular disease (CVD) is a leading cause of death in the western countries [1]. Studies have shown that impaired renal function is to be considered a risk factor in relation to CVD and patients suffering from renal disease have a higher risk of CVD [2-4]. Also, chronic kidney disease (CKD) is found to be strongly associated with an increased risk of myocardial infarction (MI) and CVD mortality [5,6]. Furthermore, CKD is found to affect patients on a global scale and with an increasing incidence and prevalence [7]. After ST-segment elevation MI (STEMI) and non-STEMI the mortality has been reported to be significantly higher among patients with renal disease compared to patients with preserved renal function [8-10]. Today primary PCI is the recommended reperfusion strategy when treating patients with STEMI, which also applies to STEMI patients with renal dysfunction [11]. Limited data are available on the outcome after primary PCI in STEMI patients with RI, because they have been underrepresented in randomized trials, as renal failure is a commonly used exclusion criterion [12].

In our study, data from the Western Denmark Heart Registry (WDHR) were used in order to assess the impact of moderate and severe renal insufficiency (RI) on short- and long-term mortality among unselected STEMI patients treated with primary PCI. Primary PCI has been the recommended treatment for STEMI after publication of the results of the DANish trial in Acute Myocardial Infarction-2 (DANAMI-2) in 2003 [13].

## Methods

### Setting and design

The study was conducted using WDHR for patients treated at Odense University Hospital. A detailed description of the databases has been reported previously [14]. The study was a registry study and ethical approval was not required.

### Patients and procedures

To be eligible for primary PCI, patients must meet the following criteria: 1) symptoms present less than 12 hours from onset of pain to time of catheterization, and 2) ST-segment elevation (at least 0.1 mV in two or more standard leads or at least 0.2 mV in two or more contiguous pre-cordial leads) or a new left bundle-branch block (LBBB). Patient with cardiogenic shock were not excluded. We used the WDHR to identify all primary PCIs performed from January 1, 2002 through December 31, 2010. Drug eluting stent (DES), bare metal stent (BMS), glycoprotein IIb/IIIa receptor blocker, and intra-aortic balloon pump was administered at the operator’s discretion. All patients received antiplatelet regimen including a bolus of 10,000 IU heparin, lifelong acetylsalicylic acid (75–150 mg once daily), and clopidogrel with a loading dose of 300 mg followed by maintenance with 75 mg daily. The recommended duration of clopidogrel treatment was 3 to 12 months until November 2002 and 12 months thereafter.

Blood samples were taken from the arterial sheath before the first contrast injection and serum creatinine - concentration was assessed in the hospital laboratory. Estimation of renal function is commonly based on estimated creatinine clearance (CrCl). Different methods are available. Studies have shown that The Modification of Diet in Renal Disease (MDRD) formula gives a reliable estimated CrCl [15-17], representing estimated glomerular filtration ratio (eGFR). Therefore the MDRD formula was used in this study: eGFR = estimated CrCl = 186 × standardized S-Cr^-1.154^ × age^-0.203^ × 0.742 [if female] [18]. The unit of this equation is expressed as mL/min per 1.73 m^2^ body surface area. An eGFR less than 60 mL/min per 1.73 m^2^ was considered equivalent to RI. Based on eGFR the study population was divided into three groups, a group with eGFR ≥ 60 mL/min per 1.73 m^2^, a group with moderate RI: CrCl ≤30 < 60 mL/min per 1.73 m^2^, and a group with severe RI: CreaCl < 30 mL/min per 1.73 m^2^. This classification of patients into different stages of CKD is identical with those universally endorsed and based on the National Kidney Foundation data [7] where patients with an eGFR ≥ 60 mL/min per 1.73 m^2^ is STAGE I/II, patients with 30 ≤ eGFR < 60 mL/min per 1.73 m^2^ are in STAGE III, and patients with eGFR < 30 mL/min per 1.73 m^2^ are in STAGE IV/V.

### Endpoints

Primary end-point of the study was all cause-mortality rate. Data on mortality were obtained from the Danish Civil Registration System [19,20], which has kept electronic records on the gender, date of birth, changes in address, date of emigration, and changes in vital status of the entire Danish population since 1968.

### Statistics

Continuous variables were presented as medians with inter quartile range (IQR 25th, 75th) or mean ± 1 standard deviations (SD). Medians were compared using the Mann–Whitney *U* test, and means were compared using the unpaired *t* test. Categorical variables were presented as numbers and percentages. Distributions of categorical variables were compared using the Chi-square test.

We counted end-point events that occurred during the follow-up period and compared rates for the two groups (CrCl ≥60 mL/min per 1.73 m^2^ vs. CrCl <60 mL/min per 1.73 m^2^). Follow-up began on the date of primary PCI procedure and continued until date of death, December 31, 2010 or after 5 years follow-up (to ensure at least 10% of the study population at risk), whichever came first. Kaplan-Meier curves for all-cause mortality according to kidney function (CrCl ≥ 60 mL/min per 1.73 m^2^, CrCl ≤30 < 60 mL/min per 1.73 m^2^ and CrCl <30 mL/min per 1.73 m^2^) were obtained.

Cox proportional hazards regression analysis was used to estimate the hazard ratio (HR) mortality. Crude and adjusted hazard ratios (HRs) with 95% confidence intervals (CIs) were computed. Potential confounders associated with time to death in the univariable Cox regression analysis were included in the multivariable Cox regression model. Thus, in the final model, we adjusted for RI, age diabetes mellitus, hypertension, previous myocardial infarction, treatment with glycoprotein IIb/IIIa receptor blocker, Killip class and duration of procedure. All data analyses were carried out using SPSS software version 20. A two-sided P value <0.05 was considered significant.

## Results

**Figure 1:**
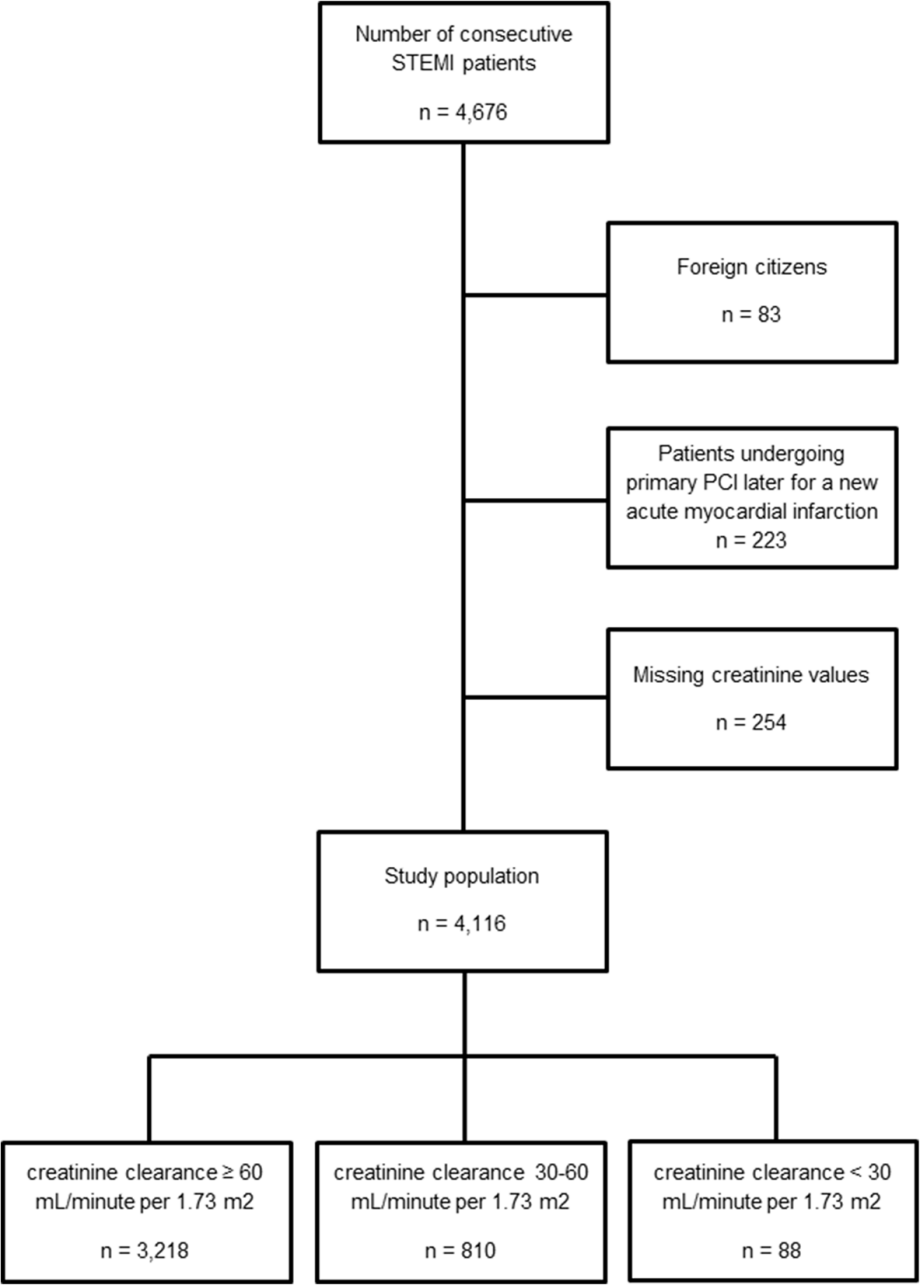
Flow diagram of participant selection.

A total of 4,676 consecutive patients were treated with primary PCI for STEMI or new onset LBBB MI at Odense University Hospital between January 1, 2002 and December 31, 2010. Mortality data was not available for 83 patients, who were foreign citizens. Patients undergoing a later acute MI after the first index procedure (n = 223) were excluded. In 254 patients the creatinine values were not available. Thus, the final study population consisted of 4,116 patients; of these were 898 patients diagnosed with RI, defined as CrCl < 60 mL/min per 1.73 m^2^, and 3,218 patients had a preserved kidney function (Figure 1).

Baseline characteristics of patients with RI and patients with a preserved kidney function are listed in Table 1. Patients with RI were older and more likely to be female, to have diabetes, hypertension, previous MI, hypercholesterolemia and less likely to be smokers. Among patients with RI, patients with severe RI (CrCl < 30 mL/min per 1.73 m^2^) more often had diabetes. Characteristics of angiographic findings, lesions and treatment procedures also differed between patients with normal and reduced CrCl, respectively. Patients with RI were more likely to have multi-vessel disease, a higher Killip class, left main culprit lesion, more complex lesions and longer duration of procedure. Patients with RI were less often treated with DES (Table 2).

**Table 1:** Baseline clinical characteristics according to kidney function

	**CrCl ≥ 60**	**Valid cases**	**CrCl < 60**	**Valid cases**	***P *** **value (CrCl ≥ 60 vs. CrCl < 60)**	**CrCl ≤30 < 60**	**CrCl < 30**	***P *** **value (CrCl < 30 vs. CrCl ≤30 < 60)**
Number of patients - no.	3,218	3,218	898	898		810	88	
Male gender - no. (%)	2,486 (77.3)	3,218	524 (58.4)	898	<0.001	471 (58.1)	53 (60.2)	0.707
Age – (year)	61.5 ± 12.1	3,218	73.5 ± 11.0	898	<0.001	73.5 ± 10.8	72.8 ± 12.8	0.532
Family history - no. (%)	1,235 (39.8)	3103	194 (23.5)	824	<0.001	178 (23.8)	16 (20.8)	0.548
Smoking - no. (%)	1,712 (57.2)	2991	290 (39.1)	741	<0.001	257 (38.1)	33 (49.3)	0.075
Body Mass Index – (kg/m^2^)	26.8 (4.4)	2,132	26.6 (4.6)	507	0.256	26.7 (4.6)	24.9 (4.2)	0.009
Diabetes - no. (%)	272 (8.5)	3218	120 (13.4)	898	<0.001	101 (12.5)	19 (21.6)	0.017
Hypertension - no. (%)	930 (29.2)	3,180	404 (46.6)	867	<0.001	358 (45.8)	46 (54.1)	0.143
Previous coronary artery bypass grafting - no. (%)	64 (2.0)	3,214	23 (2.6)	893	0.283	21 (2.6)	2 (2.3)	1.000
Previous percutaneous coronary intervention - no. (%)	203 (6.4)	3,164	58 (6.7)	868	0.778	48 (6.1)	10 (11.8)	0.048
Previous myocardial infarction - no. (%)	330 (10.4)	3,168	127 (14.5)	875	0.001	108 (13.7)	19 (13.7)	0.31
Lipid lowering therapy - no. (%)	617 (19.4)	3,173	201 (23.2)	866	0.015	173 (22.2)	28 (32.9)	0.025
Glycoprotein IIb/IIIa receptor blocker – no. (%)	1,445 (48.2)	2,998	285 (34.4)	828	<0.001	263 (35.8)	22 (26.5)	0.110
Systolic blood pressure - (mmHg)	122.9 ±24.9	2,467	118.1 ±30.5	662	<0.001	118.7 ±30.3	112.4 ±32.0	0.118
Diastolic blood pressure - (mmHg)	71.8 ±13.9	2,449	66.0 ± 15.1	654	<0.001	66.4 ± 15.1	62.3 ± 15.9	0.043

**Table 2:** Angiographic and procedural characteristics according to kidney function

	**CrCl ≥ 60**	**Valid cases**	**CrCl < 60**	**Valid cases**	***P *** **value (CreaCl ≥ 60 vs. CreaCl < 60)**	**CrCl ≤30 < 60**	**CrCl < 30**	***P *** **value (CrCl < 30 vs. CrCl ≤30 < 60)**
Number of patients - no.	3,218	3,218	898	898		810	88	
Multivessel disease no. (%)	1,304 (41.6)	3,135	520 (59.4)	876	<0.001	464 (58.7)	56 (65.9)	0.198
Infarct related artery –no. (%)		3,144		867	<0.001			0.230
Left anterior descending artery - no. (%)	1,383 (44.0)		340 (39.2)			314 (40.2)	26 (30.6)	
Left circumflex artery - no. (%)	451 (14.3)		109 (12.6)			100 (12.6)	9 (10.6)	
Right coronary artery - no. (%)	1,252 (39.8)		374 (43.1)			329 (42.1)	45 (52.9)	
Left main - no. (%)	58 (1.8)		44 (5.1)			39 (5.0)	5 (5.9)	
Anterior STEMI or LBBB – no. (%)	1,401 (45.2)	3,100	372 (43.1)	864	<0.001	343 (43.8)	29 (35.8)	0.100
Killip class – no. (%)		3,152		878	<0.001			0.001
I	2,925 (92.8)		676 (77.0)			624 (78.5)	52 (62.7)	
II	135 (4.3)		62 (7.1)			55 (6.9)	7 (8.4)	
III	51 (1.6)		51 (5.8)			46 (5.8)	5 (6.0)	
IV	41 (1.3)		89 (10.1)			70 (8.8)	19 (22.9)	
Preintervention TIMI flow – no. (%)		3,143		863	0.248			0.891
Grade 0	2,198 (54.2)		2,794 (58.7)			438 (56.2)	49 (59.9)	
Grade 1	265 (6.5)		282 (5.9)			51 (6.5)	4 (4.8)	
Grade 2	597 (14.7)		623 (13.1)			117 (15.0)	11 (13.3)	
Grade 3	978 (24.1)		1,052 (22.1)			174 (22.3)	19 (22.9)	
Final TIMI flow - no. (%)		3,143		863	<0.001			0.097
Grade 0	73 (2.3)		50 (5.8)			41 (5.3)	9 (10.8)	
Grade 1	28 (0.9)		20 (2.3)			20 (2.6)	0 (0.0)	
Grade 2	166 (5.3)		78 (9.0)			70 (9.0)	8 (9.6)	
Grade 3	2,876 (91.5)		715 (82.9)			649 (83.2)	66 (79.5)	
Lesion length – mm median (IQR)	15.00 (10.0-20.0)	3,110	15.0 (10.0-20.0)	855	0.676	15.00 (10.0-20.0)	15.00 (10.0-20.0)	0.318
Reference segment – mm median (IQR)	3.3 (3.0-3.6)	3,120	3.2 (3.0-3.5)	851	0.007	3.2 (3.0-3.5)	3.2 (2.8-3.5)	0.320
Minimum lumen diameter – mm median (IQR)	0.0 (0.0-0.2)	3,134	0.0 (0.0-0.2)	859	0.749	0.0 (0.0-0.2)	0.0 (0.0-0.2)	0.625
Sapheneous vein graft – no. (%)	10 (0.3)	3,145	4 (0.5)	867	0.526	2 (0.3)	2 (2.4)	0.007
Lesion type B2/C	1,012 (32.9)	3,082	338 (40.3)	838	<0.001	302 (39.7)	36 (46.2)	0.041
Stent length – mm median (IQR)	18.0 (14.0-23-0)	2,963	18.0 (14.0-24.0)	754	0.913	18.0 (14.0-24.0)	18.0 (13.0-24.0)	0.830
Stent number - no. (%)		3,218		898	<0.001			0.007
0	255 (7.9)		144 (16.0)			120 (14.8)	24 (27.3)	
1	2,578 (80.1)		643 (71.6)			591 (73.0)	52 (59.1)	
2+	385 (12.0)		111 (12.4)			99 (12.2)	12 (13.6)	
Drug-eluting stent – no. (%)	1,962 (62.4)	3,146	366 (41.4)	885	<0.001	338 (42.3)	28 (32.6)	0.007
Max balloon pressure – atm median (IQR)	16.0 (14.0-18.0)	3,075	15.5 (14.0-17.3)	814	0.086	15.5 (14.0-17.0)	15.5 (14.0-18.0)	0.899
Max balloon diameter – mm median (IQR)	3.6 (3.2-3.8)	3,073	3.4 (3.2-3.8)	814	0.012	3.4 (3.2-3.8)	3.3 (3.0-3.7)	0.126
Duration of procedure - minutes median (IQR)	16.0 (10.0-26.0)	3,205	17.0 (11.0-28.0)	895	0.008	17.0 (11.0-28.0)	20.0 (12.0-30.8)	0.238
Flouro time - minutes median (IQR)	6.4 (4.0-11.2)	3,174	7.7 (4.5-13.0)	878	< 0.001	7.5 (4.4-13.0)	9.0 (6.0-13.3)	0.077
Contrast – ml median (IQR)	120.0 (75.0-180.0)	877	100.0 (75.0-175.0)	3,159	0.007	120.0 (75.0-185.0)	110.0 (80.0-174.0)	0.572

STEMI: ST-segment elevation myocardial infarct.

LBBB: new onset left bundle branch block.

IQR: Interquartile range.

**Figure 2:**
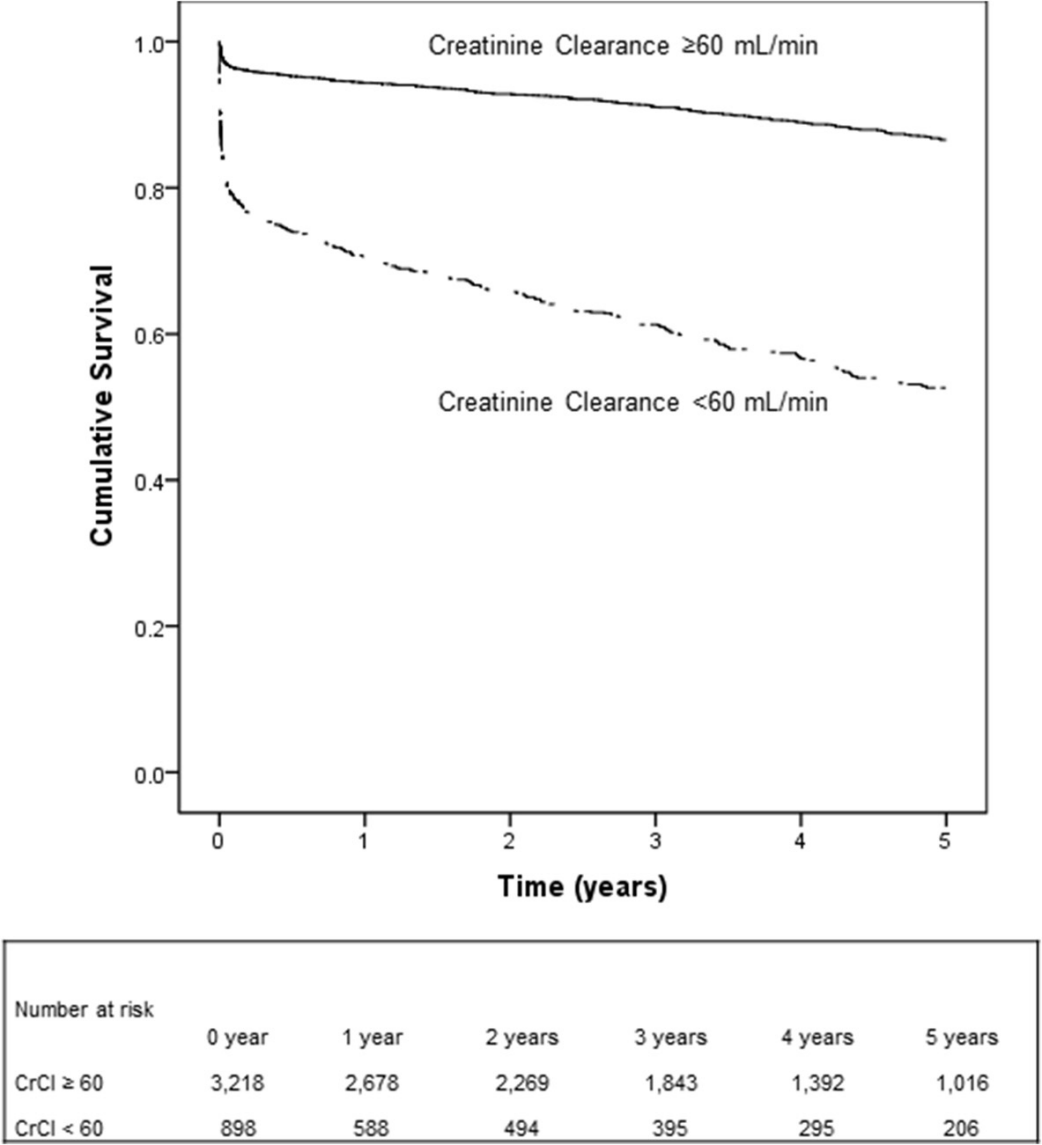
**Kaplan-Meier curves for all-cause mortality in patients with CrCl < 60 mL/min per 1.73 m**^**2 **^**and CrCl ≥ 60 mL/min per 1.73 m**^**2**^**.**

The median follow-up interval was 3.3 years (25th – 75th percentile: 1.4-5.0 years), with a 1-year mortality of 10.7% (n = 441) and 5-year mortality of 17.2% (n = 707). Among patients with a preserved kidney function and patients with RI, 30-day all-cause mortality was 3.5% (n = 112) and 20.9% (n = 188), respectively (log-rank p < 0.001); 1-year all-cause mortality was 5.7% (n = 179) and 29.4% (n = 262), respectively (log-rank p < 0.001); 5-year all-cause mortality was 13.4% (n = 328) and 47.4% (n = 379), respectively (log-rank p < 0.001). Figure 2 shows the 5-year all-cause survival of the study population when stratified into two groups with CrCl < 60 mL/min per 1.73 m^2^ and CrCl ≥ 60 mL/min per 1.73 m^2^ respectively.

**Figure 3:**
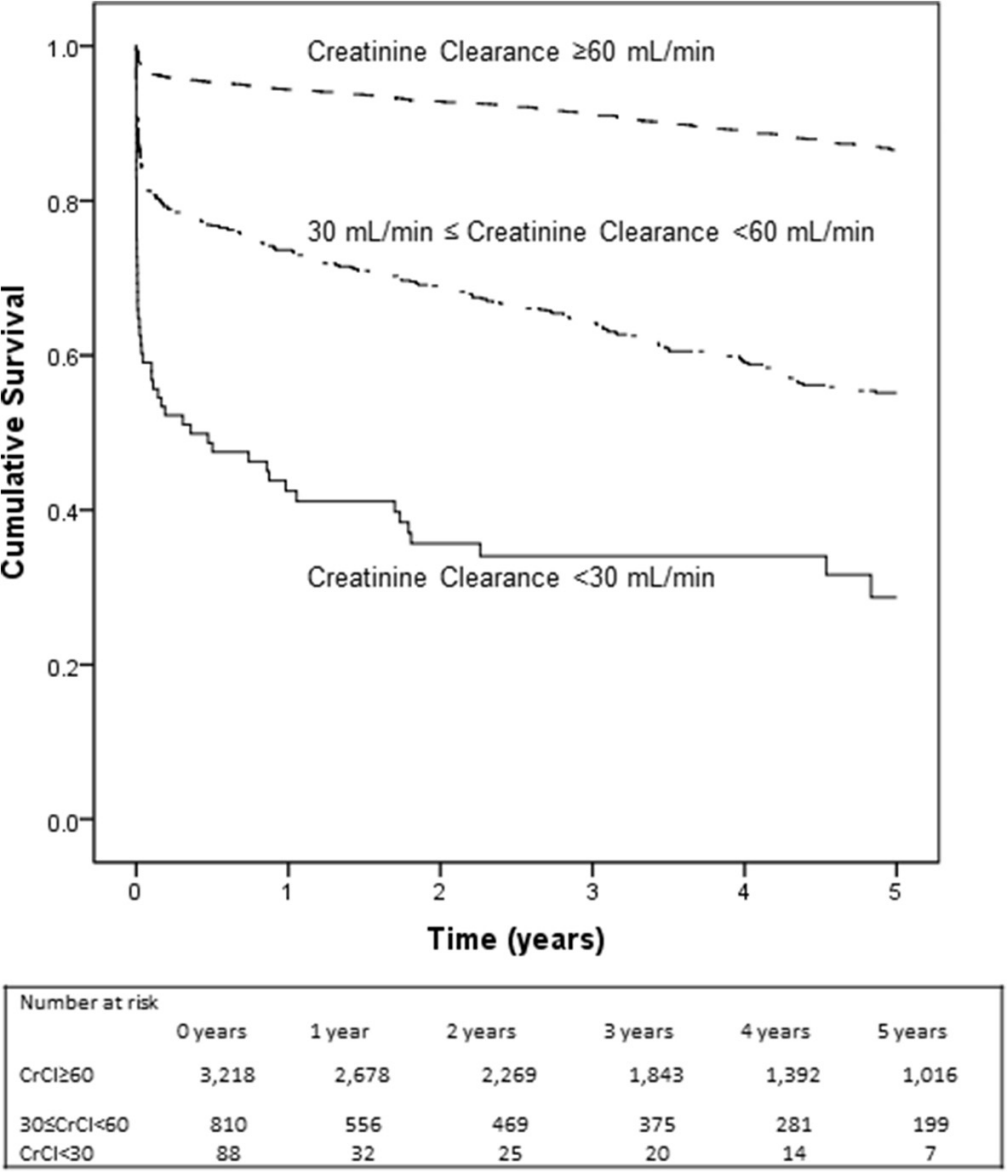
**Kaplan-Meier curves for all-cause mortality in patients with CrCl <30 mL/min per 1.73 m**^**2 **^**vs. CrCl ≤30 < 60 mL/min per 1.73 m**^**2 **^**and CrCl ≥60 mL/min per 1.73 m**^**2**^**.**

Among patients with RI (group 1: CrCl <30 mL/min per 1.73 m^2^ and group 2: CrCl ≤30 < 60 mL/min per 1.73 m^2^) the all-cause mortality rates were: at 30-day, 40.9% (n = 36) group 1 vs. 18.8% (n = 152) group 2 (log rank p < 0.001); at 1-year, 57.5% (n = 50) group 1 vs. 26.4% (n = 212) group 2 (log rank p < 0.001) and at 5-year, 71.3% (n = 58) group 1 and 44.8% (n = 321) group 2 (log rank p < 0.001). Figure 3 shows the 5-year all-cause survival of STEMI patients when stratified into three groups based on CrCl.

Table 3 shows the crude HR associated with 1-year mortality. CrCl < 60 mL/min, diabetes, hypertension, previous myocardial infarction and increasing Killip class were associated with an increased mortality. Male gender and treatment with glycoprotein IIb/IIIa receptor blocker were associated with a lower mortality.

**Table 3:** crude and adjusted hazard ratio of covariates associated with 1-year mortality in Cox regression analysis

	**Valid cases**	**Crude hazard ratio (95% CI)**	***P *** **value**	**Adjusted hazard ratio (95% CI)**	***P *** **value**
Male gender	4,116	0.68 (0.56-0.83)	<0.001	1.11 (0.88-1.41)	0.354
Creatinine clearance	4,116		<0.001		<0.001
Creatinine clearance ≥ 60 mL/min		Reference		Reference	
Creatinine clearance ≤30 < 60 mL/min		5.31 (4.35-6.49)		2.71 (2.09-3.51)	
Creatinie clearance < 30 mL/min		15.37 (11.27-21.04)		7.09 (4.82-10.44)	
Age – years	4,116	1.07 (1.06-1.08)	<0.001	1.04 (1.03-1.06)	<0.001
Diabetes mellitus	4,116	2.47 (1.95-3.13)	<0.001	1.77 (1.33-2.35)	<0.001
Hypertension	4,047	1.26 (1.03-1.53)	0.025	1.40 (1.11-1.76)	0.004
Previous myocardial infarction	4,043	1.99 (1.57-2.53)	<0.001	1.53 (1.14-2.07)	0.006
Glycoprotein IIb/IIIa receptor blocker	3,826	0.49 (0.40-0.61)	<0.001	0.68 (0.53-0.86)	<0.001
Multivessel disease	4,011	2.32 (1.91-2.83)	<0.001	1.15 (0.90-1.46)	0.265
Infarct related artery	4,011		<0.001		0.023
Right coronary artery		Reference		Reference	
Left anterior descending artery		1.19 (0.96-1.48)		1.31 (1.03-1.68)	
Left circumflex artery		1.02 (0.74-1.40)		1.24 (0.88-1.74)	
Left main		4.02 (2.73-5.92)		1.92 (1.20-3.08)	
Killip class	4,030		<0.001		<0.001
I		Reference		Reference	
II		2.32 (1.64-3.30)		1.20 (1.20-2.69)	
III		4.54 (3.17-6.51)		1.68 (1.68-3.79)	
IV		9.55 (7.32-12.46)		1.86 (2.86-5.75)	
Duration of procedure - minutes	4,100	1.01 (1.01-1.02)	<0.001	1.01 (1.00-1.01)	0.007

After adjustment for potential confounders (Table 3) we found CrCl, diabetes, age, hypertension and Killip class to be associated with increased 1-year mortality. After adjustment for covariates associated with mortality, RI was associated with increased mortality at 30-day (adjusted HR 2.38, 95% CI 1.34-4.21), 1-year (adjusted HR 2.29, 95% CI 1.50-3.50) and 5-year mortality (adjusted HR 2.02, 95% CI 1.50-2.72) compared to patients with preserved kidney function.

## Discussion

Based on an unselected cohort of STEMI patients this study aimed to explore the impact of renal disease on all-cause mortality among 4,116 STEMI patients treated with primary PCI. In this real-world setting we found, that RI was associated with increased short- and long-term mortality. The strength of the present study is that the patients are unselected, all-comer and consecutive. Today guidelines suggest, that patients with STEMI and renal dysfunction is treated in the same way as other STEMI patients with the exception of administration of contrast dye and some medications [11]. However, as primary PCI in STEMI patients is an emergent therapy; neither the kidney function nor the creatinine clearance level will be known in these patients at the time of the primary PCI. In our registry, most of the patients were diagnosed prehospital in the ambulance and referred directly to the catheterization laboratory, where the first blood sample was collected from the arterial sheath before contrast injection. In a recent study Fox et al. [12] showed from the Acute Coronary Treatment and Intervention Outcome Network (ACTION) registry, that one third of patients with STEMI had an eGFR < 60 mL/min per 1.73 m^2^. This is a higher number than the 22% of patients with RI we found in our study, which may be related to the patient population, as patients in the ACTION registry had a different comorbidity risk, as the rate of diabetes mellitus and hypertension were 2 to 3 times higher in the ACTION registry compared to our registry data. In contrast to our study, Fox et al. described that information about kidney function were available to the treating physicians when therapeutic decisions were made, and only 90% of patients with STEMI received reperfusion therapy (80% as primary PCI and 10% were treated with fibrinolysis). In our study patients with RI were more likely to receive primary PCI without stent implantation and less likely to receive treatment with glycoprotein IIb/IIIa receptor blocker compared to patients without RI. Among 2,597 STEMI and non-STEMI patients Medi et al. [21] found, that patients with RI and comorbidity were less likely to undergo coronary angiography, despite having the same frequency of primary PCI treatment as non-RI patients after angiography.

In our real-world setting registry we found, that RI was associated with increased short- and long-term mortality. This is in accordance with previous studies where RI also has been found to be associated with a higher 30-days mortality and long term mortality [22-25]. Seyfarth et al. [26] found an increase in adjusted hazard ratio after one year follow-up of 1.20 per 10-mL/min decrease in eGFR. In a study of Morel et al. [27] STEMI and non-STEMI patients were found to have a higher 9-month all-cause mortality when diagnosed with renal disease. Sadeghi et al. [28] studied 1,933 patients from the “Controlled Abciximab and Device Investigation to Lower Late Angioplasty Complications (CADILLAC)” trial, where baseline creatinine levels were obtained before angiography, including 350 patients with RI defined as CrCl < 60 mL/min per 1.73 m^2^. In our registry cohort, both short-term and 1-year mortality rate was more than twice as high as in the study from Sadeghi et al. [28]. This difference is probably caused by (1) an increased mortality risk in non-randomized and all-comer patients compared to results from a randomized controlled study and (2) that patients with cardiogenic shock were not excluded in our study. Bertomeu-Gonzales et al. [29] studied the relationship between Killip class and impact of renal disease on mortality and found, that renal disease was stronger associated with mortality in STEMI patients with Killip I compared to patients with a higher Killip class. In our study, patients with RI were more likely to be older and female and they were more likely to have diabetes, hyperlipidemia, and hypertension. The finding of women representing a large proportion of STEMI patients with RI is consistent with findings from other studies [10,28,30,31]. RI was found to be an independent predictor of 1-year mortality whereas we did not find gender to be an independent risk factor for 1-year mortality when adjusted for confounders. Similar findings have been made by Damman et al. [32] where 3-year mortality was found to be associated with a decrease in renal function, whereas gender difference was not proven to be a predictor of mortality after adjustment for confounders.

### Limitations

The validity of our findings depends on data quality and the ability to control for potential confounding. Like all observational studies, our study is prone to biases related to unmeasured factors. Bias due to unknown variables cannot be eliminated. Patients with cardiogenic shock were not excluded from the present study. We did not have systematically access to creatinine values taken in the following days after the primary PCI or information about contrast-induced nephropathy. Patients with RI were more often treated with balloon dilatation without stent implantation or bare metal stent. One explanation for this treatment strategy may be related to the more common finding of severely calcified lesions, which are known to limit complete DES expansion. We also lacked data on causes of mortality, however, in a previous STEMI cohort from Western Denmark Heart Registry, we found that especially the early causes of death was caused by a cardiac reason: the 1-year mortality reason was cardiac in 75% of the patients, whereas the 3-year mortality reason is cardiac death in 60% of the patients [33].

## Conclusion

In unselected STEMI patients treated with primary PCI, RI was associated with increased short- and long-term mortality compared to patients with a preserved kidney function.

## Competing interests

The authors declare that they have no competing interests.

## Authors’ contributions

JES and LOJ designed the study and were responsible for data management and for design and implementation of the statistical analysis. All other authors enrolled patients or contributed to data collection. JES and LOJ drafted the report, which was subsequently reviewed by all authors. All authors have seen the final submitted report and agree with its contents.

## Pre-publication history

The pre-publication history for this paper can be accessed here:

http://www.biomedcentral.com/1471-2261/14/15/prepub
